# Percutaneous cryoneurolysis for intercostal neuralgia due to bifid rib in a pediatric patient

**DOI:** 10.1007/s00247-025-06483-0

**Published:** 2025-12-12

**Authors:** Joshua Verhagen, Anna Sorensen, Sarah Tracy, Eric Monroe

**Affiliations:** 1 University of Wisconsin School of Medicine and Public Health, 1675 Highland Ave, Madison, WI 53792 USA; 2 Department of Radiology, University of Wisconsin School of Medicine and Public Health, 1675 Highland Ave, Madison, WI 53792 USA; 3 Department of Surgery, University of Wisconsin School of Medicine and Public Health, Madison, USA

**Keywords:** Cryoablation, Bifid rib, Intercostal neuralgia, Interventional radiology

## Abstract

Bifid ribs are rare congenital anomalies, usually asymptomatic and discovered incidentally on imaging. However, they can cause significant pain, posing diagnostic and therapeutic challenges, particularly in pediatric populations. We report a case of a 3-year-old female with chronic anterior chest wall pain attributed to a bifid right fourth rib. Imaging confirmed the anomaly without associated soft tissue masses. After transient symptom relief with lidocaine patches and intercostal nerve blocks, she underwent successful percutaneous image-guided cryoneurolysis of the right third to fifth intercostal nerves. The patient experienced marked pain relief post-procedure without complications, with sustained benefit at 4 months. Bifid ribs may be an under-recognized cause of intercostal neuralgia in children. Image-guided percutaneous cryoneurolysis may represent a safe and effective treatment option for symptomatic relief.

## Introduction

Congenital bifid ribs are rare skeletal anomalies, occurring in less than 1% of the population, and are typically incidental findings during chest imaging [1]. The pathogenesis of these anomalies is not entirely understood, but it is thought to result from incomplete embryologic fusion of the costal processes from the vertebral and sternal segments, with the fourth rib most commonly involved, especially on the right side and more frequently in females [2].

While most bifid ribs are asymptomatic, there are reports of localized pain and palpable chest wall masses, particularly when adjacent intercostal nerves are affected [3]. Often, the diagnosis is made incidentally; therefore, the true prevalence of this condition is unknown. In pediatric patients, these symptoms can be difficult to assess, and a broad differential should be kept in mind for a patient presenting with persistent intercostal pain.

Management of symptomatic bifid ribs lacks standardized guidelines. Options range from conservative therapies (analgesics, topical anesthetics, and nerve blocks) to more invasive interventions such as surgical resection [3]. Cryoneurolysis, a form of cryoablation that disrupts nerve conduction, has emerged as an effective tool in pain management for a variety of acute and chronic pain conditions [4]. It is increasingly used during pediatric thoracic surgeries, such as the Nuss procedure, and for trauma-related intercostal neuralgia [4, 5].

Percutaneous image-guided cryoneurolysis allows minimally invasive, outpatient or short-stay management of nerve-related pain [6, 7]. A small number of cases have demonstrated durable pain relief from cryoneurolysis ranging from weeks to months, including one report of a patient with intractable intercostal neuralgia 10 years after trauma who experienced 9 months of relief following thoracoscopic cryoneurolysis [5–8].

Here, we present the successful application of percutaneous image-guided cryoneurolysis in a child with bifid rib-associated intercostal neuralgia, adding to the limited literature on minimally invasive management of congenital chest wall anomalies in pediatrics.

## Case description

A 3-year-old female presented with persistent right anterior chest wall pain, increasing irritability, refusal to wear a car seat harness, and sleep disturbances. This irritability was first noticed around 6 months of age. Her mother also noted a palpable firm lump in the right chest area and exacerbated pain when compressed.

**Fig. 1:**
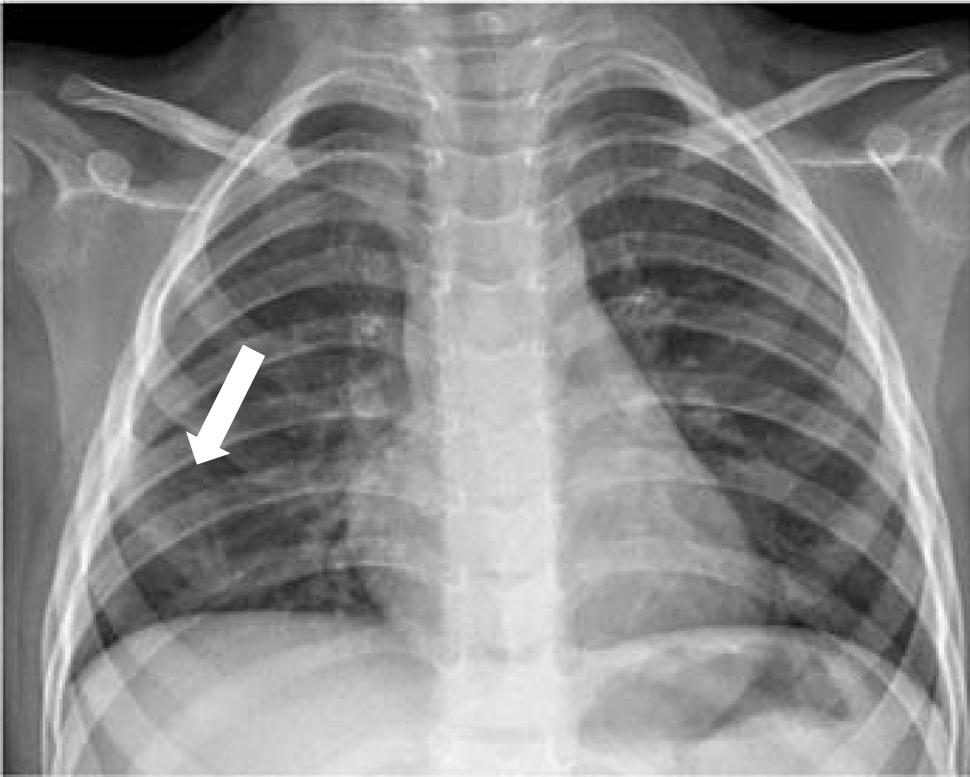
Sitting anterior-posterior chest x-ray reveals widening of the right 4th rib (*arrow*)

**Fig. 2:**
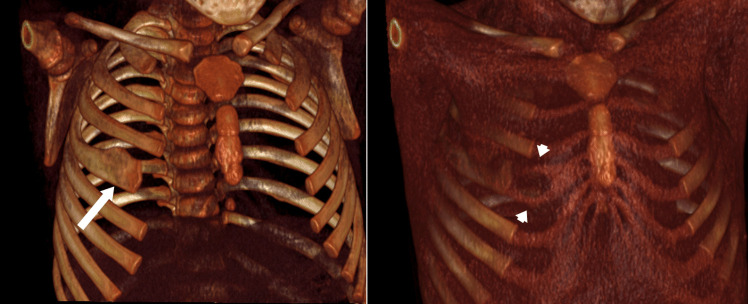
Three-dimensional CT reconstructions of the chest wall showing widening (*arrow*) of the right 4th rib without associated masses or osseous lesions. Bifurcated costal cartilage is visible in the right image (*arrowheads*)

Initial chest radiography revealed a bifid right fourth rib, as seen in Fig. 1. Subsequent non-contrast chest computed tomography (CT) confirmed bifurcation of the anterior segment of the fourth rib without associated osseous lesions or soft tissue masses. A three-dimensional reconstruction of the CT in Fig. 2 shows the bifurcated costal cartilage, with the proximal bony portion of the rib widened.

Initial symptom management involved daily application of 4% lidocaine patches, which provided partial symptom improvement but caused minor skin irritation, had to be removed for bathing and swimming, and became dislodged. To further target the pain source, an ultrasound-guided intercostal nerve block with 0.5% ropivacaine was performed at the right third and fourth intercostal spaces. This resulted in immediate but short-lived symptom resolution, with expected recurrence of pain within 24 h. This transient relief suggested a focal neuropathic component to her symptoms.

**Fig. 3:**
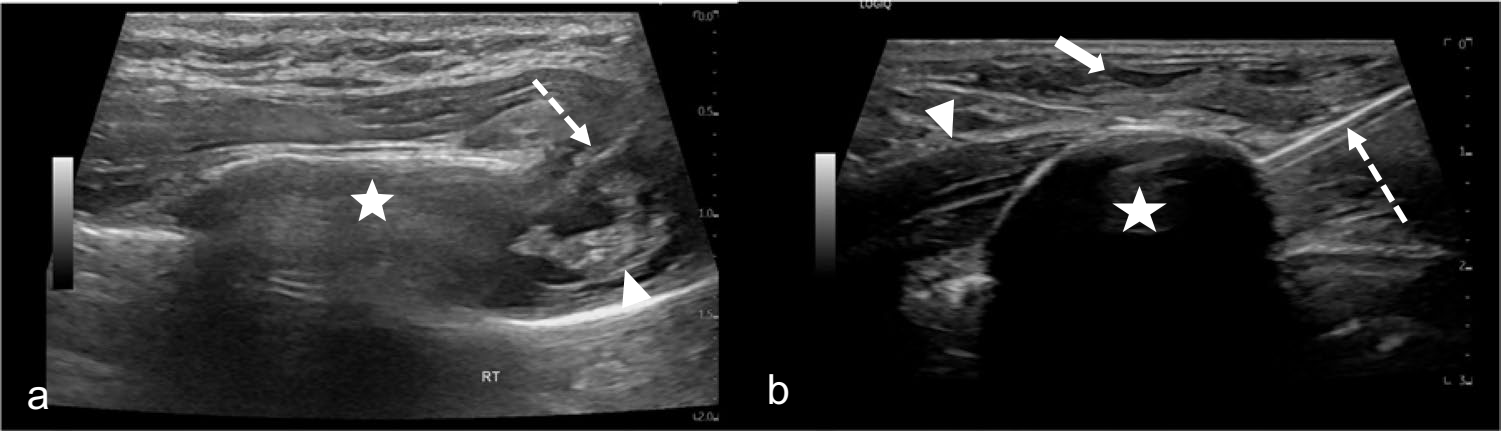
**a** Short-axis ultrasound image of the 4th rib (*star*) with needle placement (*broken arrow*) at the inferior 4th rib margin for intercostal nerve block. Hyperechoic neurovascular bundle is present along the inferior-posterior margin of the rib (*arrowhead*). **b** Long-axis ultrasound image of the 4th intercostal nerve space with hyperechoic cryoprobe (*broken arrow*) and circumscribed intramuscular hypoechoic ice ball (*star*) during the freeze cycle. Subcutaneous hydrodissection (*arrow*) and the 4th rib (*arrowhead*) are visible

Given the reproducible improvement with nerve-targeted therapy, we proceeded with definitive intercostal cryoneurolysis for sustained relief. Under general anesthesia, 3 ml of 0.5% ropivacaine was injected under ultrasound guidance along the inferior border of the right third, fourth, and fifth ribs, as shown in Fig. 3a.

**Fig. 4:**
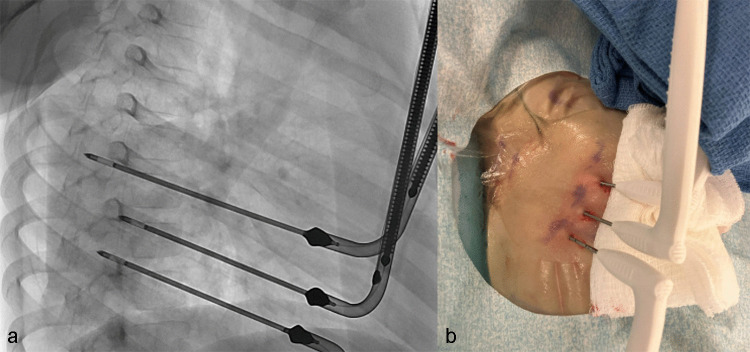
**a** Intra-procedural fluoroscopic image showing anteriorly placed cryoprobes in place at the right 3rd, 4th, and 5th intercostal spaces during cryoablation. **b** Post-procedure image of cryoprobe placements with minimal skin changes

Then, three 1.7-mm-short shaft cryoprobes (Round Ice, CryoCare, Varian, Irvine, CA) were placed percutaneously under combined ultrasound and fluoroscopic guidance at the right third, fourth, and fifth intercostal spaces to ensure coverage of the likely involved nerve segments (Fig. 4a). The cryoneurolysis protocol included simultaneous single freeze–thaw cycle, with an 8-min freeze period varying individual probe power from 25–75% throughout the period, followed by an active thaw cycle lasting 5 min. Probe placement and ice ball formation were confirmed using real-time ultrasound, as shown in Fig. 3b. Final ice ball size was determined by ultrasound with >1-cm diameter from the approximate location of the neurovascular bundle and the remaining 1 cm below the skin surface with the addition of hydrodissection. Subdermal thermal insulation was achieved with saline instillation via 25-g needles, and sterile heat packs were applied to the skin during the freeze cycle. Insulation of the lung was not performed. Post-procedure probe placement is shown in Fig. 4b.

The patient tolerated the procedure well, with no intraoperative complications, and was discharged the following day after overnight observation. One week post-procedure, the patient’s mother reported complete resolution of pain. Physical examination revealed only mild erythema at the probe insertion sites without evidence of infection.

At the 4-month follow-up, the patient continued to demonstrate sustained symptom relief, without recurrence of pain or further reliance on lidocaine patches. However, the mother reported subtle behavioral changes that started around the time of this follow-up visit. The patient became more irritable and started pressing her chest up against her mother. At her 5-month post-procedure follow-up, palpation of the deformity led to breath holding and loss of smile, indicating discomfort. The family elected to undergo a second cryoablation treatment. There were no delayed complications or skin changes following either of the ablation treatments. Given the likely return of symptoms in the future after her second ablation, the family is interested in pursuing a definitive surgical option in the future.

## Discussion

This case underscores the potential for bifid ribs to cause significant intercostal neuralgia in pediatric patients, particularly when the anomaly affects the anterior chest wall where intercostal nerves are more superficial. While well documented in adult populations, intercostal neuralgia is less commonly considered in children, where symptoms may manifest as longstanding and nonspecific [3].

Cryoneurolysis, by inducing axonotmesis via Sunderland grade II injury while sparing the surrounding connective tissue structures (endoneurium and perineurium), provides temporary disruption of nociceptive signaling with subsequent nerve regeneration [1]. Analgesia typically lasts from weeks to 3–6 months, with reports extending up to 9 months in some cases [1, 8]. The minimally invasive nature of ultrasound-guided percutaneous cryoneurolysis offers distinct advantages over surgical options, especially in pediatric populations looking to avoid surgery, including reduced recovery time, low complication rates, and avoidance of long-term pharmacotherapy [5, 6].

While evidence supporting intercostal cryoneurolysis is robust in postoperative settings such as thoracotomy and the Nuss procedure, its application in congenital anomalies such as bifid ribs remains largely unreported. This case adds to the growing body of literature suggesting that cryoneurolysis is a viable, durable option in pediatric congenital chest wall pain syndromes, avoiding the morbidity associated with surgical resection in young patients. It is primarily indicated for intercostal neuralgia refractory to conservative treatment methods.

Potential complications of intercostal cryoneurolysis primarily relate to inadequate or excessive freezing. Insufficient freeze temperatures may produce only transient analgesia due to incomplete axonal disruption (Sunderland grade I), underscoring the importance of accurate temperature monitoring and adherence to adequate freeze–thaw parameters. Conversely, excessive or poorly localized freezing can result in collateral injury to surrounding tissues, including the skin and pleura. To mitigate cutaneous injury, real-time ultrasound visualization of the ice ball is essential, combined with protective strategies such as subdermal saline hydrodissection and application of external warming packs during the freeze cycle [9]. The proximity of intercostal nerves to the pleura also raises concern for inadvertent freezing of lung tissue. This risk can be minimized by maintaining continuous imaging surveillance and monitoring ice ball extension near the pleural surface. In cases where the nerve lies in close proximity to the pleura or visualization is suboptimal, CT guidance or injection of a small volume of extrapleural air may be employed to physically displace and insulate the lung [10].

Allodynia and neuropraxia may occur if ablation parameters are subtherapeutic or if adjacent structures are inadvertently affected. Careful calibration of probe energy delivery based on nerve size, depth, and adjacent blood flow is essential to achieve an optimal balance between effective axonotmesis and tissue preservation. In pediatric patients, additional technical challenges arise due to decreased fat and muscle bulk, which can limit the containment of the ice ball and increase the risk of freezing nearby structures. Meticulous use of hydrodissection and protective measures is particularly critical in smaller children to ensure procedural safety while maintaining efficacy [11].

Although cryoneurolysis offers a minimally invasive and repeatable method for pain control, its effects are temporary, as nerve regeneration typically restores sensation and potential pain recurrence over several months. Repeat treatments may be performed as needed, potentially delaying or obviating the need for surgical intervention in young patients. For patients with persistent or refractory symptoms, surgical options such as intercostal neurectomy with implantation of the proximal nerve stump into adjacent muscle or bone have been reported as effective, long-term solutions for severe intercostal neuralgia [12]. Another alternative includes neuromodulatory approaches such as peripheral nerve stimulation, which has shown promise in adult populations for managing chronic neuropathic chest wall pain [13]. While these alternatives may be considered in select cases, percutaneous cryoneurolysis remains an attractive first-line procedural option due to its safety profile, reversibility, and capacity for outpatient management. A better understanding of the duration of symptom relief, utility of repeat treatments, and complication risks is an area for continued research.

Bifid ribs, though often incidental, can cause debilitating neuropathic chest wall pain in pediatric patients. Image-guided percutaneous cryoneurolysis is a safe, effective, and minimally invasive option for achieving sustained pain relief in patients with pain that is refractory to conservative therapy. Pediatric providers should consider intercostal neuralgia in the differential diagnosis of chronic chest wall pain, especially when associated with structural anomalies such as bifid ribs.

## Data Availability

No datasets were generated or analysed during the current study.
